# Influence of aging on the quantity and quality of human cardiac stem cells

**DOI:** 10.1038/srep22781

**Published:** 2016-03-07

**Authors:** Tamami Nakamura, Tohru Hosoyama, Daichi Kawamura, Yuriko Takeuchi, Yuya Tanaka, Makoto Samura, Koji Ueno, Arata Nishimoto, Hiroshi Kurazumi, Ryo Suzuki, Hiroshi Ito, Kensuke Sakata, Akihito Mikamo, Tao-Sheng Li, Kimikazu Hamano

**Affiliations:** 1 Department of Surgery and Clinical Science, Yamaguchi University Graduate School of Medicine, 1-1-1 Minami-kogushi, Yamaguchi, Ube 755-8505, Japan; 2 Center for Regenerative Medicine, Yamaguchi University Graduate School of Medicine, 1-1-1 Minami-kogushi, Yamaguchi, Ube 755-8505, Japan; 3 Department of Cardiovascular Surgery, Saiseikai Shimonoseki General Hospital, 8-5-1 Yasuoka, Shimonoseki, Yamaguchi 759-6603, Japan; 4 Department of Stem Cell Biology, Atomic Bomb Disease Institute, Nagasaki University, 1-12-4 Sakamoto, Nagasaki 852-8523, Japan

## Abstract

Advanced age affects various tissue-specific stem cells and decreases their regenerative ability. We therefore examined whether aging affected the quantity and quality of cardiac stem cells using cells obtained from 26 patients of various ages (from 2 to 83 years old). We collected fresh right atria and cultured cardiosphere-derived cells (CDCs), which are a type of cardiac stem cell. Then we investigated growth rate, senescence, DNA damage, and the growth factor production of CDCs. All samples yielded a sufficient number of CDCs for experiments and the cellular growth rate was not obviously associated with age. The expression of senescence-associated b-galactosidase and the DNA damage marker, gH2AX, showed a slightly higher trend in CDCs from older patients (≥65 years). The expression of *VEGF*, *HGF*, *IGF-1*, *SDF-1,* and *TGF-b* varied among samples, and the expression of these beneficial factors did not decrease with age. An *in vitro* angiogenesis assay also showed that the angiogenic potency of CDCs was not impaired, even in those from older patients. Our data suggest that the impact of age on the quantity and quality of CDCs is quite limited. These findings have important clinical implications for autologous stem cell transplantation in elderly patients.

Resident cardiac stem cells exist in adult human hearts and inherently mediate cardiogenesis and angiogenesis^1^,^2^,^3^. Recently, cardiac stem cells have been considered particularly promising for myocardial regeneration therapy. In this regard, methods for obtaining large amounts of cardiac stem cells and supporting cells (cardiosphere-derived cells, CDCs) from tiny cardiac specimens have been described^2^,^3^,^4^,^5^. These technical advances have made it possible to transplant autologous CDCs, thereby avoiding ethical or immunologic concerns. Excitingly, a first-in-human trial (CArdiospere-Derived aUtologous Stem Cells to Reverese ventricular dysfunction, or ‘CADUCEUS’) has already been completed and produced significant results^6^,^7^.

However, there are reports that tissue-specific stem cells undergo senescence and enter a dysfunctional state concomitantly with aging^8^. In bone marrow stem cells, advanced age contributes to the impairment of angiogenic potency^9^. Several reports have demonstrated that c-kit positive cardiac stem cells from aged mice and patients underwent senescence^10^,^11^. CDCs from aged mice also have shown senescent phenotype and decreased cell proliferation, expression of stem cell markers and differentiation^12^. However, the influence of aging on cardiac stem cells is not fully understood. In recent years, the prevalence of heart failure in old age has increased progressively with aging of this population^13^. Given that CDCs may be used in autologous transplantation, it is therefore vital that the influence of aging on CDCs is evaluated.

Here, we performed a head-to-head comparison of CDCs from patients of various ages by assessing multiple *in vitro* parameters including cell senescence and expression profile of growth factors. Our data provide insight into whether aged CDCs will be suitable for clinical use.

## Results

### CDC growth and phenotype

**Figure 1:**
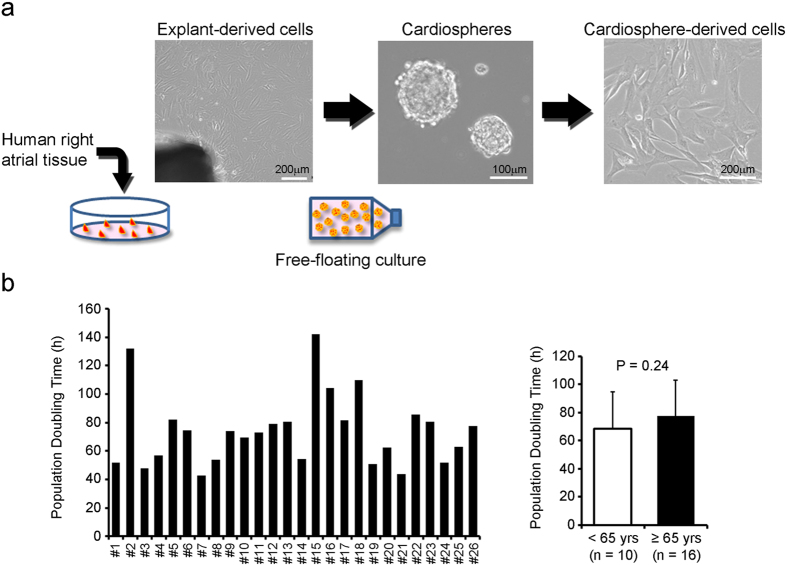
Aging is not obviously associated with growth of human CDCs. (**a**) A schematic drawing of CDC isolation. (**b**) CDCs were isolated from 26 patients with different clinical backgrounds, and the proliferative capacity of CDCs from younger (<65 yrs) and older (≥65 yrs) patients was compared. The proliferative capacity of CDCs was determined by the population doubling time from passage 0 to passage 1.

Right atrial specimens were obtained from a total of 26 patients with different clinical backgrounds. We decided the split point as 65 years, because the chronological age of 65 years as a definition of older or elderly person has been accepted in worldwide (http://www.who.int/healthinfo/survey/ageingdefnolder/en/). As shown in Table 1, the patients’ ages ranged from 2 to 83 years (median age 72.5 years) and 61.5% of them were 65 years or older. To examine CDC growth rate, population doubling time (PDT) was calculated. PDT varied between each CDC sample, and there was no significant difference between younger (<65 years) and older (≥65 years) groups (*P* = 0.24; Fig. 1b). In fact, even tiny specimens yielded a sufficient number of CDCs for experiments, regardless of age.

**Figure 2:**
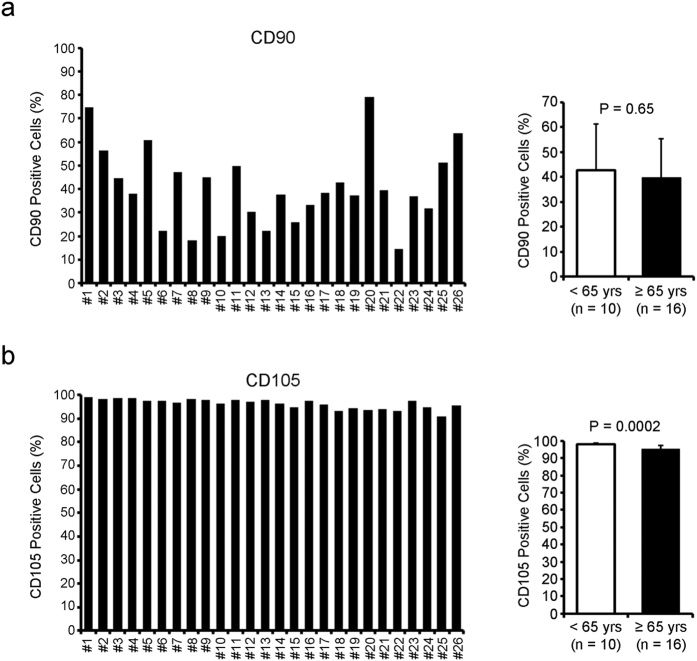
The phenotype of human CDCs varies according to the individual patient. The percentage of CD90- (**a**) and CD105-positive (**b**) cells was measured by flow cytometry. The average of CD90 and CD105 positivity was compared between CDCs from younger (<65 yrs) and older (≥65 yrs) patients.

To characterize the phenotype of CDCs, the percentage of cells positive for CD90 and CD105, which are validated markers of mesenchymal stem cells^14^, were analyzed using flow cytometry. Consistent with a previous report^15^, expression of CD105 was uniform and ranged from 90.7% to 99.0%; however, the expression of CD90 was different, and ranged from 14.4% to 79% (Fig. 2a,b). The expression of CD90 was not significantly different between the two groups (*P* = 0.65, Fig. 2a). Although the expression of CD105 in the younger group was higher than in the older group (*P* = 0.0002, Fig. 2b), the overall frequency of CD105^+^ cells was 90% or higher in all samples.

### Senescent cells in CDCs increase slightly with aging

**Figure 3:**
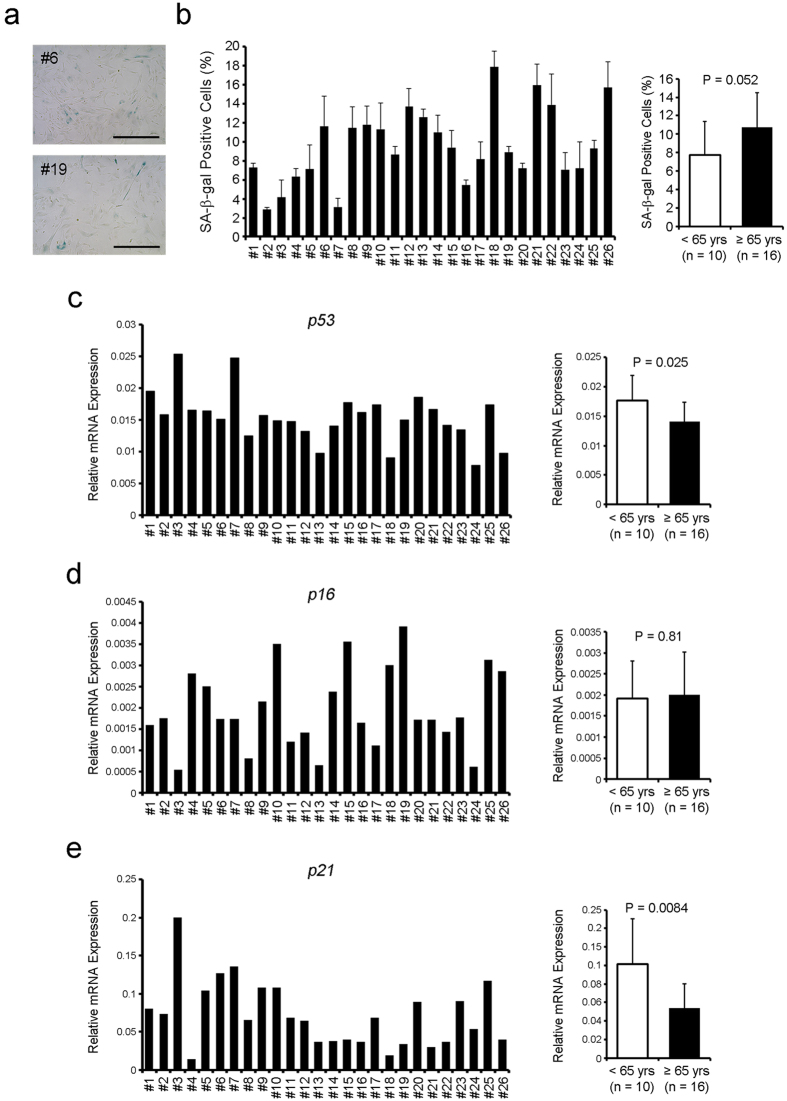
The influence of aging on human CDC senescence is limited. (**a**) Senescent cells in CDCs were identified by X-gal staining for SA-b-gal. Representative images of CDCs from younger (<65 yrs) and older (≥65 yrs) patients are shown. Blue indicates SA-β-gal-positive senescent cells. (**b**) The SA-b-gal positive cells were scored under bright-field microscopy. (**c**–**e**) Quantitative RT-PCR was performed to investigate the expression levels of mRNA encoding cell cycle inhibitors, p53, p16 and p21, which are also known as senescence markers. Scale bar shows 500 mm.

**Figure 4:**
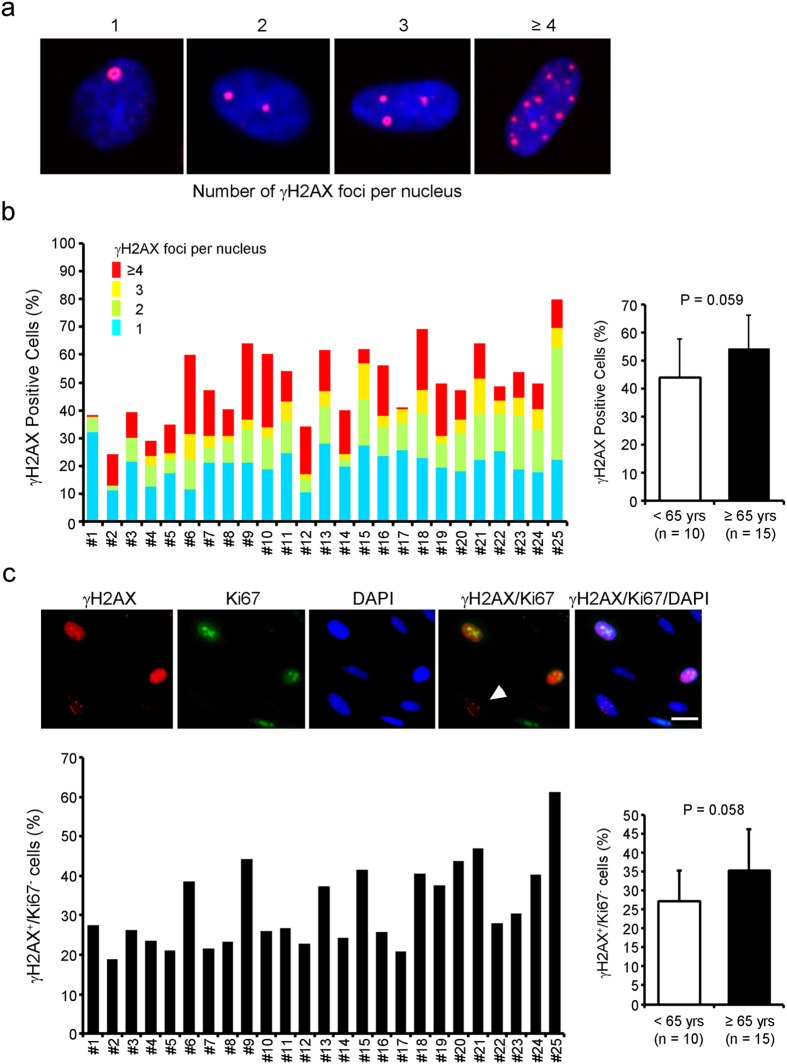
The expression of a DNA damage marker shows a slightly increasing trend in CDCs from older patients. (**a**) CDCs were classified by number of gH2AX foci per nucleus. Representative images of each CDC are shown. (**b**) The frequencies of gH2AX foci in CDCs are indicated. (**c**) The frequency of gH2AX-positive (gH2AX^+^) cells in Ki67-negative (Ki67^-^) cells in patient-derived CDCs. Arrow indicates representative gH2AX^+^ /Ki67^−^ cell. Scale bar shows 20 mm.

To investigate whether CDC senescence increased with aging, expression levels of senescence-associated β-gal (SA-β-gal) and cell cycle inhibitors (p53, p16, p21), which are validated senescence-associated markers^16^, were examined by X-gal staining and qRT-PCR, respectively. The fraction of SA-β-gal positive cells ranged 2.9% to 17.9% (mean 9.6%), and most of the CDCs did not become senescent (Fig. 3b). The frequency of SA-β-gal positive CDCs was not significantly different between the two groups, although it showed a slightly higher trend in the older patient group (*P* = 0.052, Fig. 3b). On the other hand, the mRNA levels of cell cycle inhibitors in the older group were not higher than those in the younger group (Fig. 3c–e). We also evaluated the frequency at which cells were positive for gH2AX, which is a DNA damage and senescence-associated marker (Fig. 4a). The percentage of gH2AX-positive cells showed a slightly higher trend in the older groups (*P* = 0.059, Fig. 4b). However, the number of foci per nucleus showed no association with the age of the donor (Fig. 4b). In addition, the frequency of gH2AX-positive cells in Ki67-negative cells, which is quantitative indicator of cell senescence^17^, was assessed. The percentage of them also showed a slightly higher trend in the older groups (*P* = 0.058, Fig. 4c). These results indicate that an advanced age partially influences the potential for CDCs to undergo senescence.

**Figure 5:**
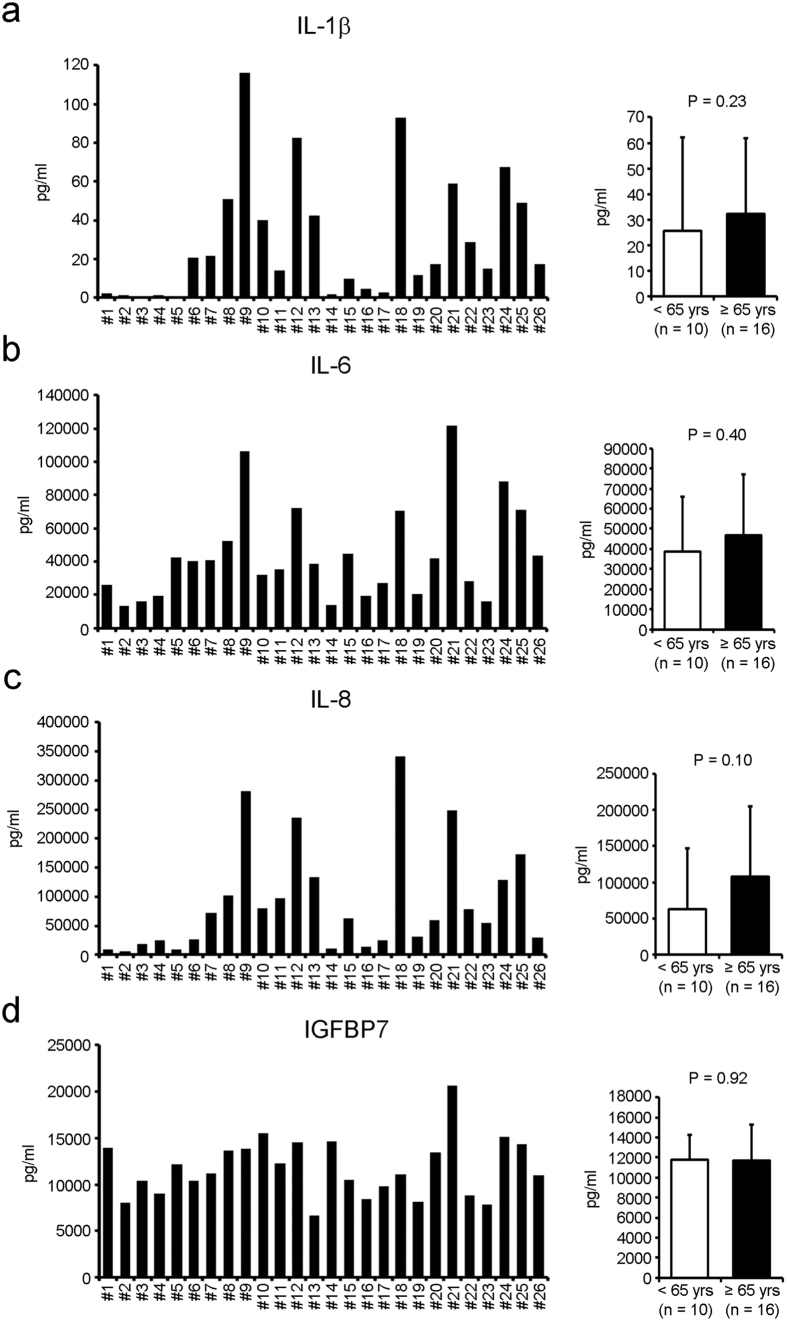
The secretion profile of senescent-associated soluble factors from human CDCs shows no significant differences between younger (<65 yrs) and older (≥65 yrs) patients. Levels of secreted senescent-associated factors including Il-1b (**a**), IL-6 (**b**), IL-8 (**c**), IGFBP7 (**d**) were assayed by ELISA.

Senescent cells are characterized by an altered secretome, termed the senescence-associated secretory phenotype (SASP)^16^. Therefore, the production of SASP factors, such as IL-1b, IL-6, IL-8 and IGFBP7 was investigated. The secretion of each factor was not significantly different between younger and older groups (Fig. 5).

### *In vitro* production of paracrine factors varies among CDCs

**Figure 6:**
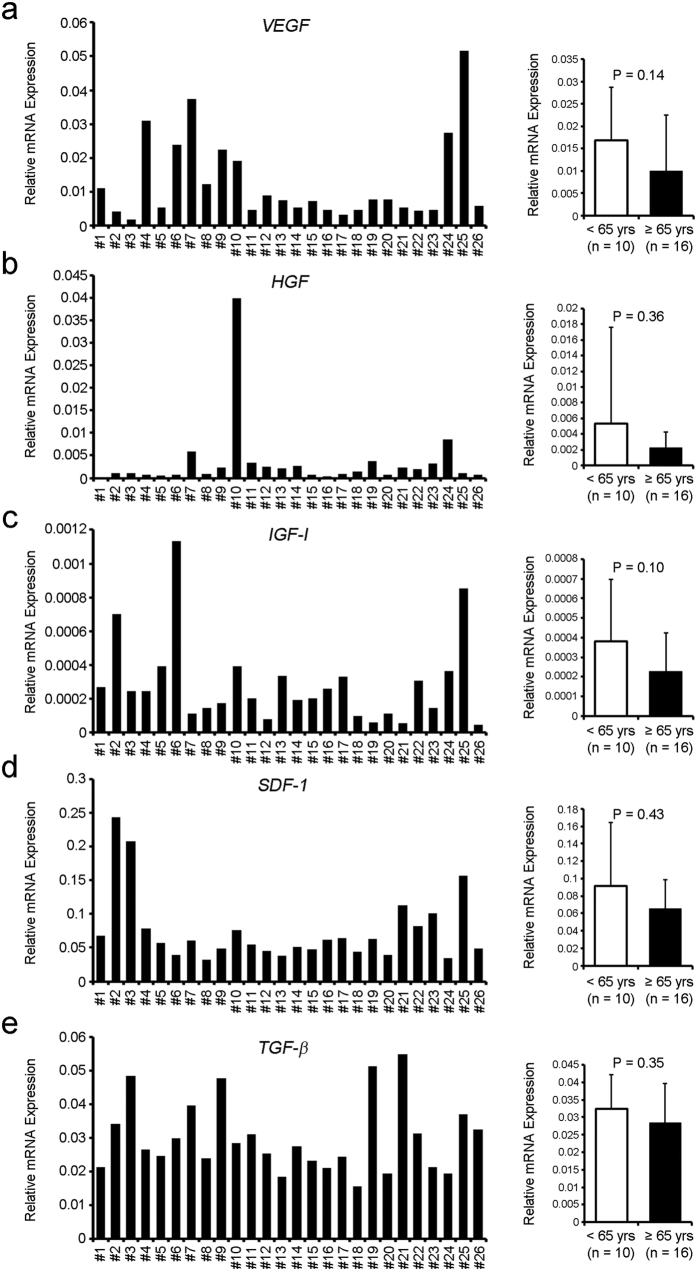
The growth factor expression profile of human CDCs shows no significant differences between younger (<65 yrs) and older (≥65 yrs) patients. The expression levels of mRNA encoding *VEGF* (**a**), *HGF* (**b**), *IGF-1* (**c**), *SDF-1* (**d**), and *TGF-b* (**e**) were investigated by quantitative RT-PCR.

There is growing appreciation that the efficacy of cell therapy depends largely on paracrine effects^18^,^19^. We thus compared the ability of CDCs to produce several growth factors (*VEGF*, *HGF*, *IGF-1*, *SDF-1*, and *TGF-β*). As shown in Fig. 6, mRNA expression levels varied among CDC samples, and no significant differences were found between the two groups (*VEGF*; *P* = 0.14, *HGF*; *P* = 0.36, *IGF-1*; *P* = 0.10, *SDF-1*; *P* = 0.43, *TGF-β*; *P* = 0.35). The secretion of VEGF, HGF and IGF-1 was also investigated, and each factor did not decrease in older group (Supplementary Figure S1).

**Figure 7:**
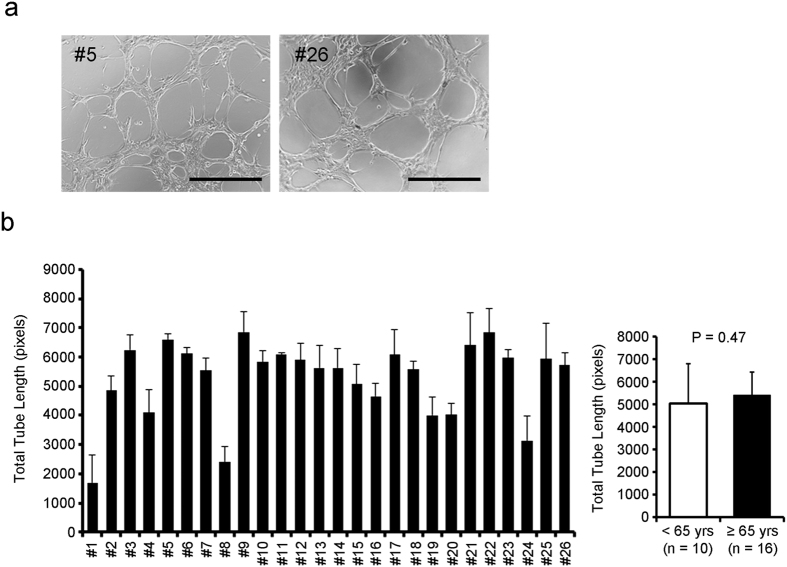
Angiogenic potential of human CDCs is not obviously associated with patient age. Tube formation assay of CDCs grown in Matrigel was performed. (**a**) Representative images of tube formation by CDCs from younger (<65 yrs) and older (≥65 yrs) patients are shown. (**b**) Total tube length per field of CDCs is shown. Scale bar shows 500 mm.

To evaluate the angiogenic potential of CDCs, we used an *in vitro* tube formation assay (Fig. 7). CDCs themselves can robustly form capillary networks (so called tubes)^20^; therefore, we used CDCs (rather than the standard human umbilical vein endothelial cells) for the tube formation assay. With the exception of a few samples (#1, #8, #24), CDCs formed tubes efficiently (Fig. 7b). The total tube length varied among CDCs, and no significant difference was recognized between the two groups (*P* = 0.47, Fig. 7b). In addition, migration ability of CDCs, which is mediated by VEGF, HGF and SDF-1^21^,^22^, was not significantly different between two groups (*P* = 0.48, Supplementary Figure S2). These results suggest that the paracrine effects of CDCs vary between individuals, and there was no statistical trend between paracrine effects and age.

## Discussion

Here, we tested whether the quantity and quality of CDCs were impaired as donor age increased. We found that the expression of senescence-associated markers, SA-β-gal staining and gH2AX showed slightly higher trend in CDCs from older patients. This suggests that CDCs from older patients are more prone to undergo senescence than those isolated from younger patients. However, the quantity and quality of CDCs assessed by cell growth, the expression of growth factors, and angiogenic ability varied among CDCs from different patients and was not obviously associated with patients’ ages. Our data suggest that in patients with different clinical backgrounds, age is not a critical determinant of the quantity and quality of CDCs.

Senescent cells accumulate in tissues with advancing age. Tissue-specific stem cells, for example those of the hematopoietic and musculoskeletal system are also known to undergo degenerative changes with age^9^,^23^,^24^, which compromises their regenerative capacity. However, few studies have focused on the influence of donor age on CDC function^12^. This prompted us to examine the function of CDCs obtained from both young and elderly patients.

First, we investigated the quantity of CDCs. For cardiac stem cell transplantation, it is important to know whether a sufficient number of CDCs can be obtained from small amounts of tissue. In contrast to murine CDCs^12^, the growth rate of human CDCs was not obviously associated with age in our study, suggesting that other factors within the heterogeneous clinical backgrounds, rather than age, may determine proliferation. Then we assessed the phenotypic characteristics of CDCs obtained from each sample, because CDCs are a natural mixture of stromal, mesenchymal, and progenitor cells. This is favors regenerative capacity^20^ and, in turn, the percentage of each cellular population may affect the therapeutic effect. In fact, a recent study showed that CDC implantation with populations expressing higher levels of CD90 was associated with reduced therapeutic effect due to the elevation of inflammatory cytokines; in contrast, CD90-negative CDCs induced a remarkable therapeutic effect^15^. Thus, we used our CD90 expression data to conclude that the therapeutic effect varied among CDCs in terms of phenotypic characteristics.

Next, we investigated the quality of CDCs by assessing cell senescence, the expression of growth factors, and *in vitro* angiogenic potency. Since no single marker is sufficient to identify cell senescence, combinations are usually used to establish the phenotype^16^. The results of SA-b-gal staining and gH2AX suggested that senescence in CDCs slightly increased with aging (Supplementary Figure S3). However, the result of SA-b-gal staining also showed that even CDCs from elderly patients, most of cells did not become senescent. Therefore we conclude that the influence of age is minimal, at least in early passage CDCs. Recent evidence suggests that cell-based therapy improves cardiac function largely via paracrine mechanisms^18^,^25^. VEGF, HGF, IGF-1, and SDF-1 play central roles in paracrine effects by mediating angiogenesis, anti-apoptosis, and recruitment of stem cells^25^. TGF-β, which is an anti-inflammatory cytokine, promotes fibrosis by activating fibroblasts in addition to promoting angiogenesis^25^,^26^. In this study, these beneficial factors did not decline with age. In addition, the angiogenic ability evaluated by tube formation assay also supported these results. Our data suggests that donor age is not a critical determinant of regenerative ability via paracrine effects.

Although we assumed that CDC function would deteriorate with age, our results actually show that the effects of age on CDCs were limited. One possible explanation is that patients’ clinical backgrounds (such as their cardiac function or the presence of diabetes mellitus) might affect CDC function. In fact, the clinical characteristics of patients in our study were quite diverse, as shown in Table 1. In a previous study, our group reported that, in addition to advanced age, the angiogenic potential of bone marrow stem cells was impaired by renal failure and anemia^9^. Human cardiac stem cells also showed that chronic heart failure negatively affected the function of cardiac stem cells^10^,^27^. However, contrary to these reports, CDCs from advanced heart failure patients showed augmented regenerative ability through an SDF-1-mediated mechanism^28^. Therefore, it remains unclear that how these patients’ factors affect the function of cardiac stem cells. In addition, these factors do not necessarily influence the quantity and quality of stem cells uniformly. For example, although hematopoietic stem cells from aged mice can proliferate, their abilities to reconstitute blood and to engraft following transplantation were impaired^29^. Therefore, it is quite possible that these factors (rather than age) are the predominant determinants of CDC function. Further studies are required to determine the critical factors that affect the regenerative ability of CDCs in the patient population. Isolation of such factors would have important clinical implications for autologous transplantation therapy of CDCs.

Another explanation may be that aged CDCs are eliminated during culture, since it takes about 1.5 months to obtain passage 2 CDCs. It is also possible that some kind of rejuvenation mechanism exists during the culture of CDCs. CDCs are obtained through a three-dimensional culture process that produces structures called cardiospheres. This niche-like structure enhances stemness via several mechanisms, such as increasing the expression of growth factors, adhesion molecules, and extracellular matrix^2^,^30^. Thus, the cardiosphere process may favor a regenerative mechanism and this process may be associated with CDC rejuvenation. However, further studies are required to confirm these speculations.

In conclusion, we found that the quantity and quality of CDCs varied between patients of diverse ages. However, the influence of age on the quantity and quality of CDCs was limited. Although we did not determine the critical patients’ factor for regenerative ability of CDCs, many factors other than age might change the quantity and quality of CDCs. Therefore, these results give some messages that the elderly patients should not be excluded from application of autologous cell transplantation therapy only by their age. In addition, we should take the specific clinical background of patients into consideration when conducting CDC-based therapy.

## Materials and Methods

### Ethics statement

All protocols were approved by the ethics review board for clinical research at Yamaguchi University (No. H22-29-4). All investigations were conducted in accordance with the Declaration of Helsinki. Informed written consent for participation in the study was obtained from all patients.

### Isolation and culture of CDCs from human samples

Human tissues were derived from right atrial biopsies belonging to 26 patients who underwent heart surgery. Human CDCs were expanded as described^3^,^5^ with some modifications. Biopsies were minced into small fragments and cultured as explants on dishes coated with 25 μg/ml of fibronectin (Corning). After about 20 days, they were harvested and seeded in 30 mg/ml of poly 2-hydroxyethyl methacrylate (Sigma-Aldrich) coated flasks to form cardiospheres. These cardiospheres were finally reseeded on fibronectin-coated dishes and grown into monolayers as cardiosphere-derived cells (CDCs) (Fig. 1a). Twice-passaged CDCs were used for experiments, except as indicated.

### Cell growth

The population doubling time (PDT) was used as an estimate of cell cycle time. PDT was calculated using the following equations: PDT = CT/Log (N/N_0_) × 3.31, where N is the final number of cells, N_0_ is the initial number of cells and CT is the time in culture^31^. PDT of CDCs was determined between passage 0 and passage 1. Cell counts were conducted using a manual hemocytometer with the trypan blue exclusion test to verify the viability of the cells.

### Senescence-associated b-galactosidase staining

CDCs were seeded on fibronectin-coated dishes and senescence-associated b-galactosidase staining was performed using the Senescence Detection Kit (BioVision, Inc.) according to the manufacturer’s protocol. The SA-β-gal-positive cells were counted under a microscope.

### Flow cytometry

CDCs were harvested as single-cell suspensions using TrypLE™ Express (Thermo Fisher Scientific). Cells were then incubated with PE-conjugated mouse anti-human CD105 antibody (# 12-1057-42, eBioscience) or FITC-conjugated mouse anti-human CD90 antibody (# 11-0909-42, eBioscience) for 30 min. The percentages of CD105 and CD90 were quantitatively measured using a Cytomics FC500 instrument with FC500 CXP Cytometer software (Beckman Coulter Co.).

### Immunostaining

CDCs cultured on 24-well culture dishes were fixed and blocked with Protein Block Serum-Free Ready-to-Use (Dako) for 1 h. Then they were incubated with rabbit monoclonal antibody against gH2AX antibody (Ser 139, #9718, Cell Signaling Technologies) for 1 h at room temperature. Then they were washed and incubated with a DyLight 550-conjugated goat anti rabbit IgG antibody (ab96884, Abcam). Next, they were incubated with rabbit monoclonal antibody against Ki67 antibody conjugated with Alexa Fluor 488 (ab197234, Abcam) for 1 h at room temperature. Nuclei were stained with DAPI. Positively stained cells were counted by using BZ-X710 All-in-One fluorescence microscope (KEYENCE). CDCs in each sample were classified by the number of gH2AX foci per nucleus (gH2AX foci per nucleus = 1, 2, 3 or ≥4) and the number of cells belonging to each category was counted. Also, the number of gH2AX positive cells in Ki67-negative cells was counted. In this particular experiment, only samples #1 to #25 were used, and #26 was not scored.

### Enzyme-linked immunosorbent assay (ELISA)

To assess the production of senescence-associated factors and growth factors, conditioned medium was collected from the human CDC cultures, and enzyme-linked immunosorbent assay (ELISA) was performed, targeted at interleukin-1b (IL-1β: R&D systems), interleukin-6 (IL-6: R&D systems), interleukin-8 (IL-8: R&D systems), insulin-like growth factor binding protein 7 (IGFBP7: Abnova corporation), vascular endothelial growth factor (VEGF: R&D systems), hepatocyte growth factor (HGF: R&D systems), and insulin-like growth factor-I (IGF-I) (R&D systems) according to the manufacturer’s protocol.

### Quantitative RT-PCR

Total RNA of CDCs was isolated using RNAeasy Mini Kit (QIAGEN). The extracted total RNA was reverse-transcribed into single-stranded cDNA using PrimeScript RT Master Mix (Perfect Real Time) kit (Takara Bio). Real-time PCR was performed using cDNA with QuantiTect SYBR Green PCR Kit (QIAGEN). Primer sequences are listed in Supplementary Table S1 32,33,34,35,36,37,38,39,40. The reaction condition was 95 °C for 15 min, and followed by 48 cycles of the following reaction: 95 °C for 10 s and 60 °C for 30 s. The quantitative PCR was performed with LightCycler software version 3.5 (Roche Applied Science) and data were evaluated using the 2^−ΔΔCT^ method.

### *In vitro* tube formation

The tube formation assay was performed as described previously^20^,^41^. Briefly, CDCs were seeded on 96-well plate coated with Matrigel^®^ (Corning) at 2 × 10^4^ cells/well. Images of forming tubes were captured 6 h later. The total tube lengths per field were measured using Angiogenesis Analyzer for ImageJ software (National Institutes of Health).

### Migration assay

To evaluate migration ability of CDCs, scratch assay was performed as described previously^42^. In 24-well culture dishes at high confluence, scratches were created using 1000 μl tips. Phase contrast images of the scratches were acquired using BZ-X710 All-in-One fluorescence microscope (KEYENCE) at 0 h and 12 h after incubation. The area of wounds was measured by using BZ-X Analyzer software (KEYENCE), and wound closure rates were calculated.

### Statistical analysis

The data were processed using Stata version 12.0 software. Data were analyzed for normal distribution and statistical significance between two groups was determined by using a two-tailed unpaired *t* test or Mann-Whitney test, as appropriate. Pearson product-moment correlation coefficient was used to correlate age and each parameter. Differences were considered as significant when *P* < 0.05.

## Additional Information

**How to cite this article**: Nakamura, T. *et al.* Influence of aging on the quantity and quality of human cardiac stem cells. *Sci. Rep.* **6**, 22781; doi: 10.1038/srep22781 (2016).

## Supplementary Material

Supplementary Information

## Figures and Tables

**Table 1:** Patient characteristics.

Case	Age (yrs)	Sex	Diagnosis	NYHA	EF (%)	HT	D	DL	Coronary disease
#1	2	M	Atrial septal defect	–	76	No	No	No	No
#2	5	M	Atrial septal defect	I	82	No	No	No	No
#3	10	F	Atrial septal defect	I	80	No	No	No	No
#4	18	M	Atrial septal defect	I	84	No	No	No	No
#5	32	M	Aortic regurgitation	II	45	No	No	No	No
#6	38	M	Aortic regurgitation	I	61	Yes	Yes	Yes	No
#7	43	M	Lone atrial fibrillation, Left atrial thrombus	I	70	Yes	No	No	No
#8	53	M	Aortic stenosis	II	74	No	No	No	No
#9	58	M	Endocardial cushion defect	II	55	Yes	No	Yes	Yes
#10	64	F	Mitral regurgitation, Tricuspid regurgitation, Atrial fibrillation	III	74	Yes	No	No	No
#11	65	F	Aortic regurgitation	II	45	Yes	No	Yes	No
#12	72	M	Prosthetic aortic valve dysfunction	II	75	Yes	No	No	No
#13	72	M	Mitral regurgitation Tricuspid regurgitation, Atrial fibrillation	II	80	Yes	No	No	No
#14	73	F	Chronic type A aortic dissection	I	83	Yes	Yes	Yes	Yes
#15	73	F	Mitral regurgitation, Tricuspid regurgitation, Aortic regurgitation	I	80	Yes	No	Yes	No
#16	75	M	Aortic stenosis	I	60	No	No	No	No
#17	76	F	Mitral regurgitation	I	75	Yes	No	No	No
#18	76	M	Thoracic aortic aneurysm	I	70	No	No	No	No
#19	77	M	Aortic stenosis	II	52	Yes	Yes	No	No
#20	78	F	Aortic stenosis	II	77	Yes	Yes	Yes	Yes
#21	79	M	Thoracic aortic aneurysm	I	70	Yes	No	Yes	Yes
#22	79	M	Thoracic aortic aneurysm	I	68	Yes	No	Yes	No
#23	81	F	Aortic stenosis	II	80	Yes	No	Yes	No
#24	83	M	Thoracic aortic aneurysm, Aortic stenosis	I	75	No	No	No	No
#25	83	F	Aortic stenosis	II	70	Yes	No	Yes	No
#26	83	F	Aortic stenosis	II	40	Yes	No	Yes	No
