# Supplementary material for: Influence of aging on the quantity and quality of human cardiac stem cells
Source: Sci Rep. 2016 Mar 7;6:22781. doi: 10.1038/srep22781 (PMC4780032; doi:10.1038/srep22781)
Supplement: Supplementary Information [file srep22781-s1.pdf]

Supporting online materials for:

Influence of Aging on the Quantity and Quality of Human Cardiac Stem Cells

Tamami Nakamura, Tohru Hosoyama, Daichi Kawamura, Yuriko Takeuchi, Yuya Tanaka, Makoto Samura, Koji Ueno, Arata Nishimoto, Hiroshi Kurazumi, Ryo Suzuki, Hiroshi Ito, Kensuke Sakata, Tao-Sheng Li, Akihito Mikamo, and Kimikazu Hamano

To whom correspondence should be addressed to:
Tohru Hosoyama (E-mail: toruhoso@yamaguchi-u.ac.jp)

This PDF file includes: Supplementary Figure S1, Supplementary Figure S2, Supplementary Figure S3, and Supplementary Table S1.

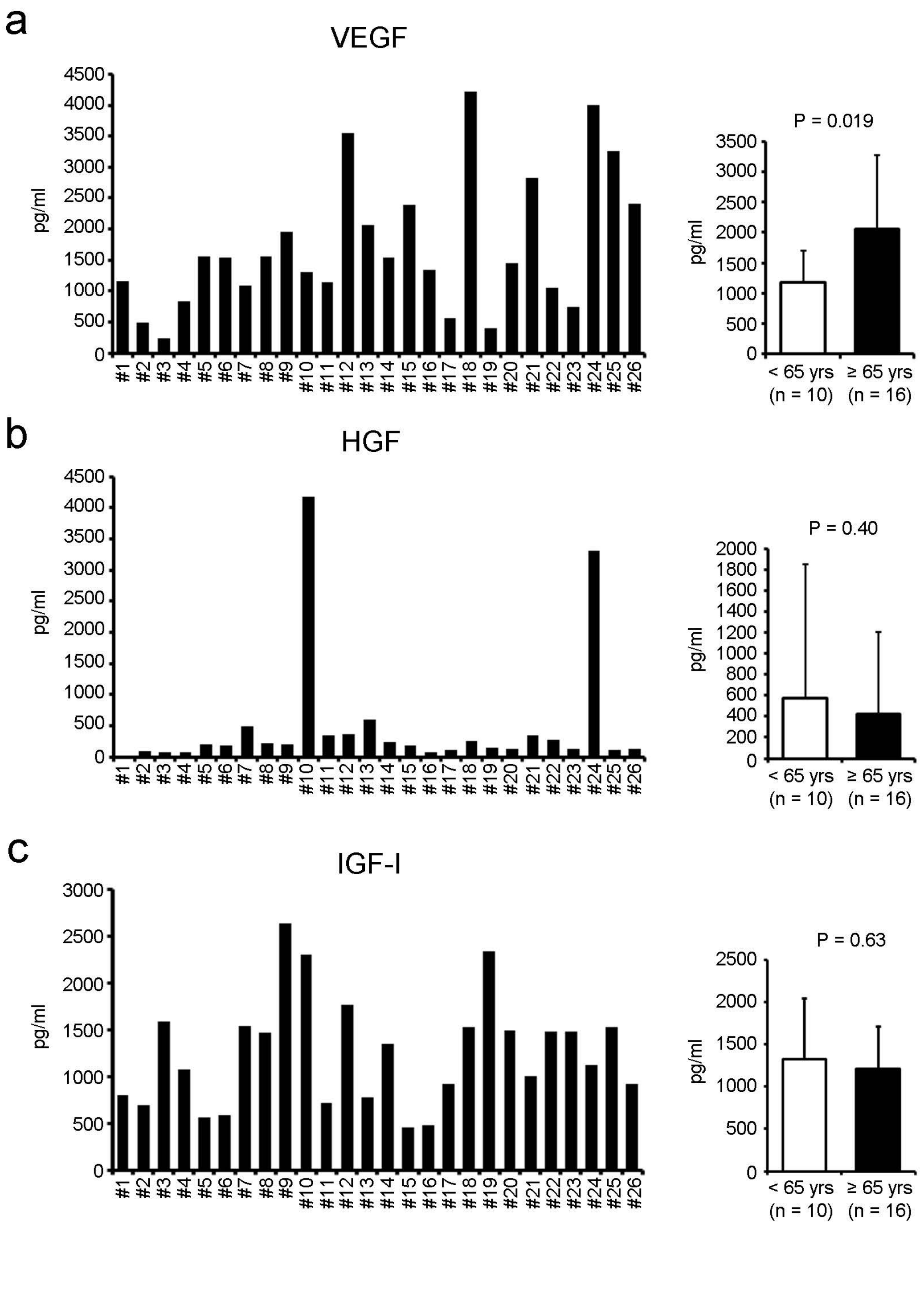

Supplementary Figure S1. The secretome analysis of CDCs. The level of VEGF (a), HGF (b), and IGF-I (c) in the medium from CDCs culture was assayed by ELISA.

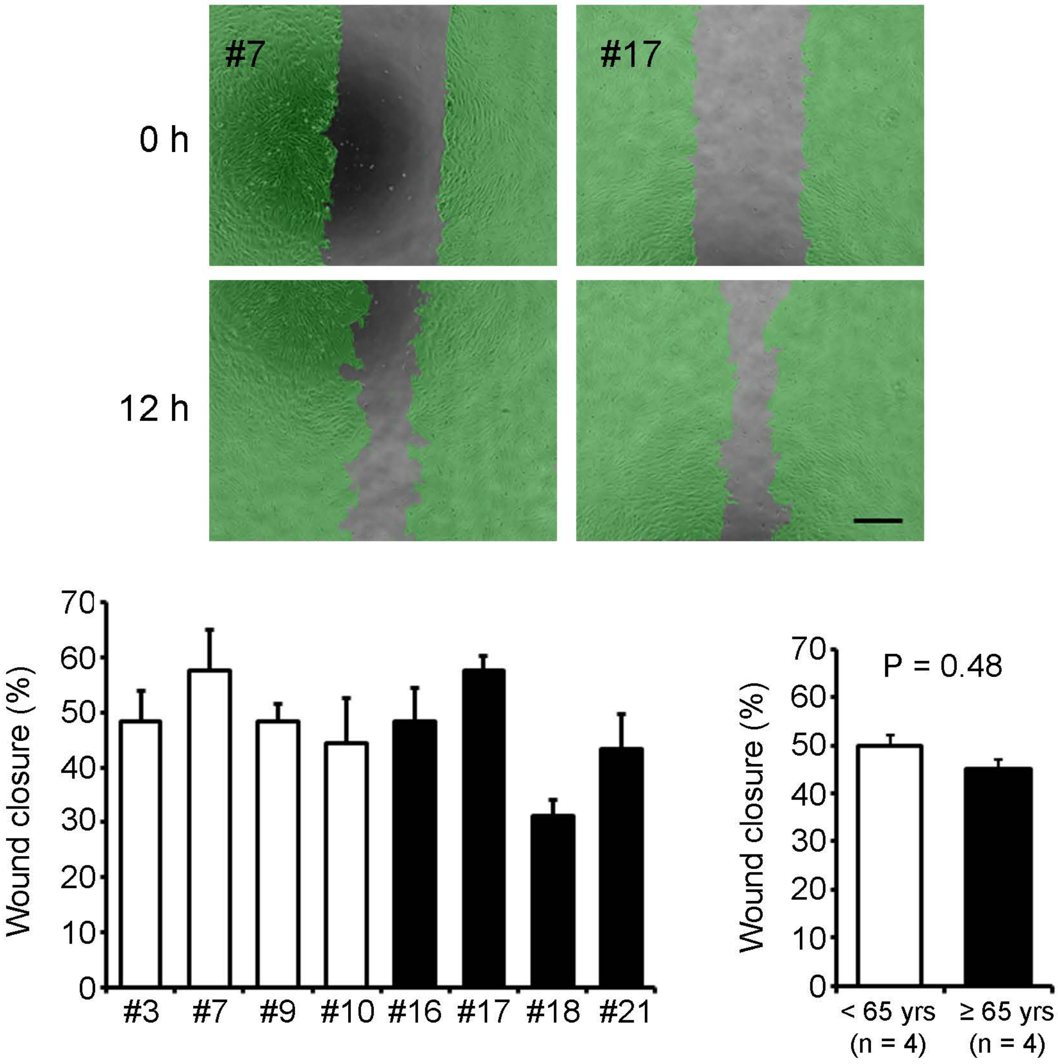

Supplementary Figure S2. Migration capacity of CDCs were analyzed by the scratch assay. Confluent CDC cultures (#7: < 65 years age, #17: ≥ 65 years age) were scraped to make about 1.2-mm of scratch and further cultured for 12 hours (Upper images). Images were virtually pigmented by green to visualize cell area. Wound closure was measured and compared between groups of < 65 years age and ≥ 65 years age (Lower graphs). Scale bar = 500 μm.

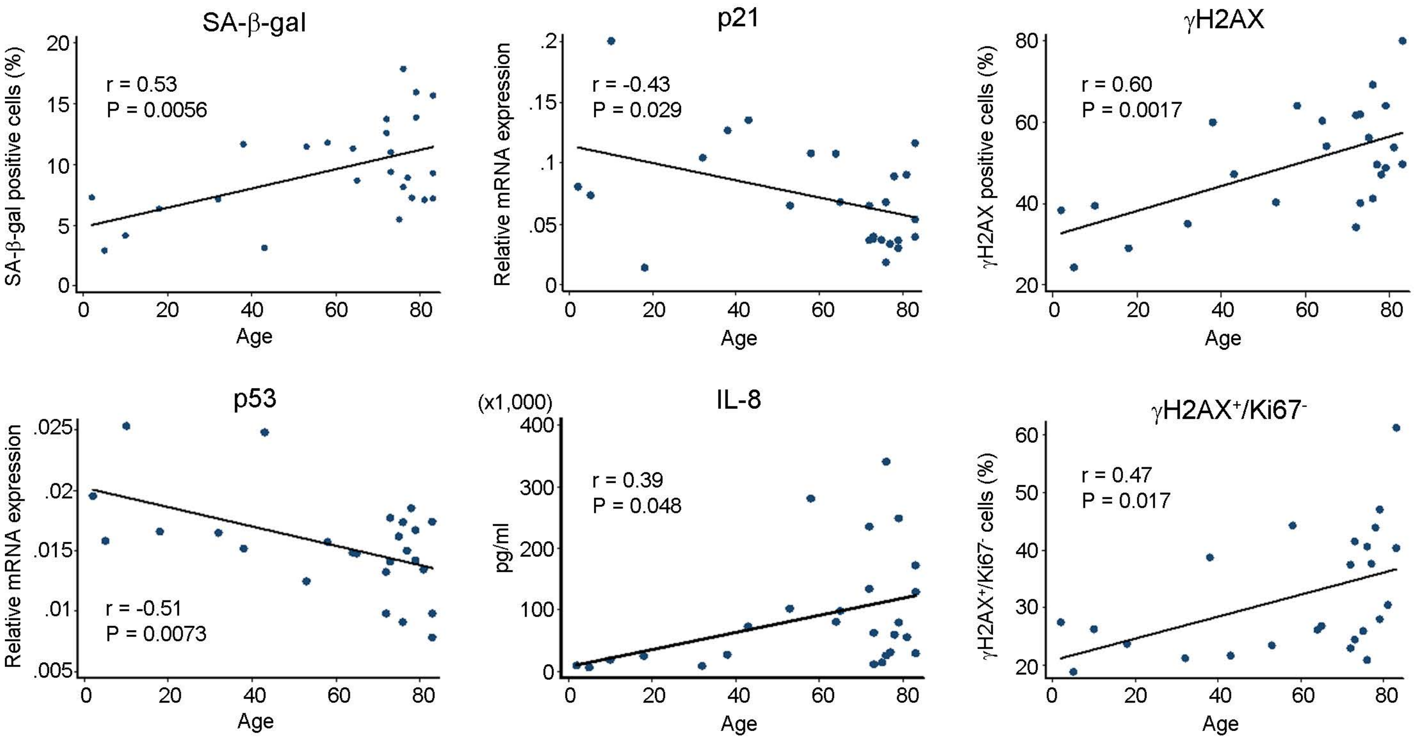

Supplementary Figure S3. A correlation analysis between age and parameters. Parameters including SA-β-gal positivity, p21 mRNA expression, p53 mRNA expression, IL-8 level, γH2AX positivity, and a distribution of γH2AX^{+} cells in Ki67^{-} cells were applied to a correlation analysis to age.

Supplementary Table S1. List of primers.

Transcript	Forword (5'-3')	Reverse (5'-3')	Reference
Ki-67	GAGGTGTGCAGAAAATCCAAA	CTGTCCCTATGACTTCTGGTTGT	(32)
p53	GCAGCGCCTCACAACCTCCG	TGATTCCACACCCCCGCCCG	(33)
p16	AGCATGGAGCCTTCGGCTGA	CCATCATCATGACCTGGATCG	(33)
p21	CACCGAGACACCACTGGAGG	GAGAAGATCAGCCGGCGTTT	(34)
VEGF	AGCCTTGCCTTGCTGCTCTA	GTGCTGGCCTTGGTGAGG	(35)
HGF	CTGGTTCCCCTTCAATAGCA	CTCCAGGGCTGACATTTGAT	(36)
IGF-1	TCTGCACGAGTTACCTGTTA	CAATCTACCAACTCCAGGAC	(37)
SDF-1	AGAGCCAACGTCAAGCATCT	CTTTAGCTTCGGGTCAATGC	(38)
TGF-β	CAACGAAATCTATGACAAGTTCAAGCAG	CTTCTCGGAGCTCTGATGTG	(39)
GAPDH	GTGGACCTGACCTGCCGTCT	GGAGGAGTGGGTGTCGCTGT	(40)
